# Characterization of outdoor air pollution from solid fuel combustion in Xuanwei and Fuyuan, a rural region of China

**DOI:** 10.1038/s41598-020-68229-2

**Published:** 2020-07-09

**Authors:** Wei Hu, George Downward, Jason Y. Y. Wong, Boris Reiss, Nathaniel Rothman, Lützen Portengen, Jihua Li, Rena R. Jones, Yunchao Huang, Kaiyun Yang, Ying Chen, Jun Xu, Jun He, Bryan Bassig, Wei Jie Seow, H. Dean Hosgood, Linlin Zhang, Guoping Wu, Fusheng Wei, Roel Vermeulen, Qing Lan

**Affiliations:** 1 Division of Cancer Epidemiology and Genetics, Occupational and Environmental Epidemiology Branch, National Cancer Institute, National Institutes of Health, 9609 Medical Center Drive, Rockville, MD 20850 USA; 2 Institute for Risk Assessment Sciences, Division of Environmental Epidemiology, Utrecht University, Utrecht, 3508 TD The Netherlands; 3 Mel and Enid Zuckerman College of Public Health, University of Arizona, Tucson, AZ 85724 USA; 4 Qujing Center for Disease Control and Prevention, Qujing, 655000 Yunnan China; 5 Department of Cardiothoracic Surgery, The Third Affiliated Hospital of Kunming Medical University (Yunnan Cancer Hospital, Yunnan Cancer Center), Kunming, 650118 China; 6 School of Public Health, Li Ka Shing, Faculty of Medicine, The University of Hong Kong, Hong Kong, SAR China; 7 Saw Swee Hock School of Public Health, National University of Singapore, Singapore, 119077 Singapore; 8 Department of Epidemiology and Population Health, Albert Einstein College of Medicine, Bronx, NY 10461-1900 USA; 9 China National Environmental Monitoring Center, Beijing, 100012 China

**Keywords:** Environmental impact, Risk factors

## Abstract

Outdoor air pollution is a growing public health concern, particularly in urban settings. However, there are limited epidemiological data on outdoor air pollution in rural areas with substantial levels of air pollution attributed to solid fuel burning for household cooking and heating. Xuanwei and Fuyuan are rural counties in China where the domestic combustion of locally sourced bituminous (“smoky”) coal has been associated with the highest lung cancer rates in China. We previously assessed indoor and personal air pollution exposures in this area; however, the influence of indoor coal combustion and household ventilation on outdoor air pollution has not been assessed. Therefore, we measured outdoor fine particulate matter (PM_2.5_), species of polycyclic aromatic hydrocarbons (PAHs) including naphthalene (NAP) and the known carcinogen benzo(a)pyrene (BaP), sulfur dioxide (SO_2_), and nitrogen dioxide (NO_2_) over two consecutive 24-h sampling periods in 29 villages. Just over half of the villages were revisited two to nine months after the initial sampling period to repeat all measurements. The overall geometric mean (GM) of outdoor PM_2.5_, BaP, NAP, and NO_2_ were 45.3 µg/m^3^, 9.7 ng/m^3^, 707.7 ng/m^3^, and 91.5 µg/m^3^, respectively. Using linear mixed effects models, we found that burning smoky coal was associated with higher outdoor BaP concentrations [GM ratio (GMR) = 2.79] and lower outdoor SO_2_ detection rates (GMR = 0.43), compared to areas burning smokeless coal. Areas with predominantly ventilated stoves (> 50% of stoves) had higher outdoor BaP (GMR = 1.49) compared to areas with fewer ventilated stoves. These results show that outdoor air pollution in a rural region of China was associated with the type of coal used for cooking and heating indoors and the presence of stove ventilation. Our findings suggest that efforts of household stove improvement to reduce indoor air pollution have resulted in higher outdoor air pollution levels. Further reducing adverse health effects in rural villages from household coal combustion will require the use of cleaner fuel types.

## Introduction

Outdoor air pollution is a major environmental health concern that was linked to 3.7 million deaths worldwide in 2012 and 4.2 million deaths in 2016 (six percent due to lung cancer)^1–3^. An upward trend in attributable deaths from 1990 to 2015 was partially due to increasing outdoor air pollution in low- and middle-income countries^4^. Outdoor air pollution is considered as a leading environmental cause of lung cancer by the International Agency for Research on Cancer (IARC) which recently classified outdoor air pollution and particulate matter (PM) as a Group 1 carcinogen (carcinogenic to humans)^5–7^.

While automobile traffic is the predominant source of outdoor air pollution in developed urban areas, this is not the case in rural, underdeveloped areas where local sources such as cooking and heating stoves contribute significantly to air pollution levels^8^. Whereas many epidemiological studies focus on the health impacts of outdoor air pollution in urban areas, outdoor air pollution and its adverse health effects in rural areas are often overlooked^8^. More than 60% of the Chinese population lives in rural regions. In this population, household air pollution (HAP) due to domestic combustion of solid fuels contributes significantly to the total burden of disease^9^; however, epidemiological data on outdoor air pollution exposure in rural China is limited. Xuanwei and Fuyuan are rural counties located in Southwestern China that have elevated rates of nonmalignant and malignant lung diseases including the highest lung cancer rate in China that is directly associated with HAP from bituminous (“smoky”) coal^10,11^. We previously reported indoor and personal exposure levels to PM_2.5_, polycyclic aromatic hydrocarbons (PAHs), black carbon, NO_2_, and SO_2_ from burning solid fuels in Xuanwei and Fuyuan^12–15^; and reported outdoor measurements of black carbon were positively correlated with the level of indoor measurements^15^. Additionally, we previously reported that there was variation in lung cancer risk for specific subtypes of smoky coal mined from different Xuanwei geological coal deposits^16,17^, while improving home ventilation by installing stoves with chimneys and converting to portable stoves was associated with both a reduction in lung cancer rates and specific HAP constituents in this region^18,19^. However, the contribution to neighborhood-level outdoor air pollution from both household ventilation and the indoor burning of coal mined from various coal deposits has not been assessed in this region. Given the clear need for outdoor air pollution research in rural settings, especially within China, we evaluated outdoor air pollution concentrations and the potential factors associated with those levels in Xuanwei and Fuyuan. As a part of a comprehensive evaluation of air quality study in Xuanwei and Fuyuan, this paper characterizes the outdoor air pollution in a rural area with higher incidence of lung cancer in China and provides quantitative evidence concerning the necessity of changing to clean fuel energy.

## Results

**Figure 1:**
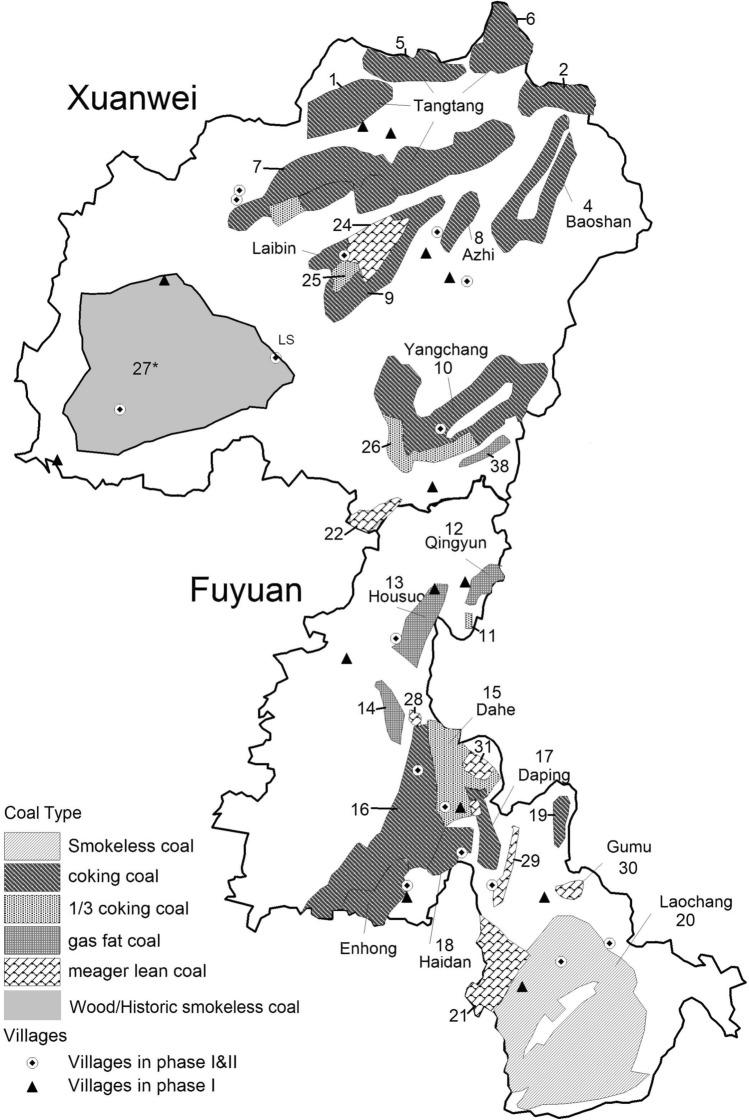
Map of geological coal deposits and study villages in Xuanwei and Fuyuan counties. Classification of coal regions based on the State Standard of China Coal Classification (GB5751-86); 1/3 coking, coking, gas fat, and meager lean coals are subtypes of smoky coal. *Historic smokeless coal deposit. Figure adapted from Fig. 1 in “Lung cancer risk by geologic coal deposits: A case–control study of female never-smokers from Xuanwei and Fuyuan, China” by Wong et al.^17^. Adapted with permission.

Figure 1 shows the distribution of the 29 villages (16 of them were visited at both Phases I and II) and coal mine regions in Xuanwei and Fuyuan counties. Most villages were located in smoky coal mine areas in the center, east, north, and south parts of Xuanwei. Several villages in southwestern Xuanwei were in a historically smokeless coal mine region (27), where farmers have changed to wood. In Fuyuan, smokeless coal mines are predominantly located in the south, while smoky coal mines are in the north.

### Background information of villages

Background measurement information about the villages and meteorological factors are summarized in Table 1. Less than 14% of the villages were within 5 km of a known factory, mine, or power station. The average village population size was 840 (750 SD). In the past, smoky coal was used in areas of Xuanwei and Fuyuan that had undergone stove improvement interventions, including the installation of chimneys, in an effort to reduce HAP^18,19^. As a result, more than half of all households in 22 of the 29 villages (75.9%) included stoves ventilated with chimneys.

**Table 1:** Environmental and meteorological characteristics of the villages.

Characteristics	N (k)^a^	AM (SD)	Median (25%,75% percentile)
Daily average outside temperature, °C	88 (29)	10.7 (5.8)	11.2 (5.6,16.4)
Daily average outside humidity, %	88 (29)	78 (16.8)	82.2 (69.9,90.0)
Population	29 (29)	840 (750)	570 (420,920)
**Season**^b^			
Autumn	33 (17)		
Spring	25 (13)		
Summer	2 (1)		
Winter	28 (15)		
**Proportion of chimney stoves in a village, k (%)**			
> 50%	22 (75.9)		
≤ 50%	7 (24.1)		
**Factory within 5 km, k (%)**			
Yes	4 (13.8)		
No	21 (72.4)		
Unknown	4 (13.8)		
**Coal mine within 5 km, k (%)**			
Yes	4 (13.8)		
No	21 (72.4)		
Unknown	4 (13.8)		
**Power plants within 5 km, k (%)**			
Yes	3 (10.3)		
No	22 (75.9)		
Unknown	4 (13.8)		

*AM* arithmetic mean, *SD* standard deviation, *km* kilometer.

^a^N = the number of measurements; k = the number of villages.

^b^Measurements in 16 villages were performed across multiple seasons.

### Outdoor pollution in coal deposits

Table 2 shows outdoor measurements by coal deposit. The overall GM of outdoor PM_2.5_, BaP, NAP, and NO_2_ were 45.3 µg/m^3^, 9.7 ng/m^3^, 708 ng/m^3^, and 91.5 µg/m^3^ respectively. The overall detection rate of SO_2_ was 26.1%. Outdoor pollutant concentrations or detection rates (%Detect) were similar between the two counties [GM(GSD): 51.6(1.7) and 40.2(2.0) µg/m^3^ for PM_2.5_; 10.5(1.7) and 8.9(1.6) ng/m^3^ for BaP; 730(2.3) and 678(2.6) ng/m^3^ for NAP; 93.2(1.4) and 89.7(1.5) µg/m^3^ for NO_2_, and %Detect: 34.8 and 34.1% for SO_2_, in Xuanwei and Fuyuan, respectively]. Based on an ANOVA test there is significant variation within each coal deposit for PM_2.5_ absorbance (PM_abs_) (p < 0.05). A Tukey HSD test further reveals that PM_abs_ in villages located in several smoky coal deposits [GM(GSD): 5.8(1.7)—7.7(1.3) × 10^–5^/m] were significantly higher compared with villages located in smokeless coal deposits [GM(GSD): 3.3(1.4) × 10^–5^/m] (p < 0.05).

**Table 2:** Concentrations of outdoor air pollutants in villages of Xuanwei and Fuyuan by coal deposit.

Coal deposit in region	PM_2.5_ (µg/m^3^)			BaP (ng/m^3^)			NAP (ng/m^3^)			SO_2_		NO_2_ (µg/m^3^)			PM_abs_ (10^–5^/m)^†^		
	N	AM^a^	GM (GSD)^b^	N	AM	GM (GSD)	N	AM	GM (GSD)	N	%Detect	N	AM	GM (GSD)	N	AM	GM (GSD)
Overall	85	53.1	45.3 (1.8)	51	11.1	9.7 (1.7)	58	1,045.8	707.7 (2.4)	88	26.1	87	97.6	91.5 (1.4)	66	5.5	5.0 (1.6)
**Xuanwei**	41	58.1	51.6 (1.7)	28	12.1	10.5 (1.7)	34	1,078.8	729.9 (2.3)	44	34.8	44	97.5	93.2 (1.4)	34	6.6	6.1 (1.6)
1, 2, 4, 7, 8^%^	20	56.8	47.4 (1.8)	14	12.7	10.8 (1.8)	20	1,058.4	813.6 (2.2)	22	18.2	22	101.7	97.8 (1.3)	18	6.4	5.8 (1.7)^c^
9^%^	4	70.1	67.9 (1.3)	4	13.7	12.0 (1.8)	4	629.0	620.0 (1.2)	4	25.0	4	118.5	116.8 (1.2)	4	7.2	6.9 (1.4)^c^
10^%^	4	59.1	57.9 (1.3)	2	19.8	19.8 (1.0)	0	–	–	4	50.0	4	113.0	107.5 (1.4)	4	7.9	7.7 (1.3)^c^
LS*^,%^	4	49.2	47.8 (1.3)	3	6.2	6.1 (1.2)	4	2,282.6	682.8 (5.3)	4	25.0	4	85.1	83.7 (1.2)	2	5.9	5.8 (1.2)
**Fuyuan**	44	48.5	40.2 (2.0)	23	9.9	8.9 (1.6)	24	999.2	677.5 (2.6)	44	34.1	43	97.7	89.7 (1.5)	32	4.2	4.0 (1.4)
12, 13, 14, 38^%^	10	58.8	50.8 (1.8)	8	9.2	8.7 (1.5)	8	1,272.8	1,093.9 (1.9)	10	20.0	9	88.9	87.6 (1.2)	4	7.2	6.6 (1.6)^c^
16, 17, 19^%^	22	48.5	42.1 (1.8)	13	11.0	10.1 (1.6)	9	1,276.0	827.1 (3.2)	22	27.3	22	97.5	92.0 (1.5)	18	4.9	4.7 (1.3)
20, 27**^,%^	21	48.3	39.0 (2.2)	7	8.4	7.4(1.7)	13	475.2	413.0 (1.7)	22	31.8	22	92.8	81.7 (1.6)	16	3.5	3.3 (1.4)

^a^*AM* arithmetic mean; ^b^*GM* geometric mean, *GSD* geometric standard deviation; ^c^p < 0.05 when compared with deposit 20, 27 deposits via Tukey HSD test. *LS is located in deposit 27 in Xuanwei; **Deposit 27 is located in Xuanwei; ^%^Deposit numbers refer to the map locations of each coal source shown in Fig. 1. *NAP* naphthalene, *BaP* Benzo(a)pyrene, *SO*_*2*_ sulfur dioxide, *NO*_*2*_ nitrogen dioxide; ^†^p < 0.05 among coal deposits via ANOVA test, data were published in reference^15^.

### Outdoor pollution associated with coal type, ventilation, and season

Table 3 shows the concentrations of outdoor pollutants associated with coal type, stove ventilation, and season. Outdoor BaP and NAP concentrations in villages using smoky coal were significantly higher than those using smokeless coal [smoky coal vs smokeless coal, GM(GSD): 10.2(1.7) vs 5.7(1.4) ng/m^3^ for BaP, 795(2.4) vs 303(1.6) ng/m^3^ for NAP]; and p < 0.05 via Tukey HSD test. Further, PM_2.5_ concentrations were not significantly higher in these villages [smoky coal vs smokeless coal, GM(GSD): 47.7(1.7) vs 35.0(2.4) µg/m^3^]. However, using smoky coal was associated with significantly lower outdoor SO_2_%Detect compared with using smokeless coal (50.0% vs 21.6%; p < 0.05). Overall, villages with greater than 50% of the households using chimneys generally had higher outdoor PM_2.5_, BaP, NAP, and NO_2_ compared to those with ≤ 50% chimney use, although these differences were not statistically significant.

**Table 3:** Concentrations of outdoor air pollutants in villages of Xuanwei and Fuyuan, by coal type, stove type, and season.

	PM_2.5_ (µg/m^3^)			BaP (ng/m^3^)			NAP (ng/m^3^)			SO_2_		NO_2_ (µg/m^3^)		
	N	AM^a^	GM (GSD)^b^	N	AM^a^	GM (GSD)^b^	N	AM^a^	GM (GSD)^b^	N	%Detect	N	AM^a^	GM (GSD)^b^
**Coal type from coalmines**														
Smokeless coal	14	46.1	35.0 (2.4)	4	5.9	5.7 (1.4)	7	331	303 (1.6)	14	21.6	14	106.3	90.8 (1.7)
Smoky coal	71	54.5	47.7 (1.7)	47	11.6	10.2 (1.7)^c^	51	1,144	795 (2.4)^c^	74	50.0^d^	73	95.9	91.6 (1.4)
**Proportion of ventilated stoves in a village**														
≤ 50%	19	52.1	44.4 (2.0)	9	8.7	8.1 (1.5)	15	666	543 (1.9)	20	30.0	20	89.9	84.5 (1.4)
> 50%	66	53.4	45.6 (1.8)	42	11.6	10.1 (1.7)	43	1,178	776 (2.5)	68	25.0	67	99.9	93.7 (1.4)
**Season**														
Autumn	32	42.3	35.3 (1.9)	16	7.9	7.4 (1.4)	28	763.3	547.4 (2.3)	33	30.3	33	80.7	77.0 (1.4)
Spring	25	51.2	43.9 (1.9)	21	13.4	11.9 (1.7)^e^	17	813.9	629.2 (2.1)	24	24.0	24	105.4	97.9 (1.4)^e^
Summer	0	–	–	0	–	–	0	–	–	2	0.0	2	81.1	76.3 (1.6)
Winter	28	67.2	62.2 (1.5)^e^	14	11.3	9.9 (1.7)	13	1957.7	1,435.5 (2.3)^e,f^	28	25.0	28	112.0	107.1 (1.4)^e^

*NAP*, naphthalene, *BaP* Benzo-a-pyrene, *SO*_*2*_ sulfur dioxide, *NO*_*2*_ nitrogen dioxide.

^a^*AM* arithmetic mean. ^b^*GM* geometric mean, *GSD* geometric standard deviation. ^c^p < 0.05 when compared with smokeless coal via Tukey HSD test. ^d^p < 0.05 when compared with smokeless coal via Fisher’s exact test. ^e^p < 0.05 when compared with autumn via Tukey HSD test. ^f^p < 0.05 when compared with spring via Tukey HSD test.

ANOVA testing revealed significant seasonal variation in concentrations of the four outdoor pollutants (Table 3, p < 0.05). Concentrations of PM_2.5_, NAP, BaP, and NO_2_ were lowest in autumn. PM_2.5_ levels were 1.8 times as high in winter than in autumn (GM: 62.2 vs 35.3 µg/m^3^, p < 0.05). NAP was significantly higher in winter compared with either autumn or spring. Outdoor BaP in spring was the highest (GM: 11.9 ng/m^3^, p < 0.05) compared with levels in the autumn (GM: 7.4 ng/m^3^). Compared with levels in autumn (GM: 77.0 µg/m^3^), NO_2_ levels were significantly higher during the spring and winter (GM: 97.9 and 107.1 µg/m^3^, respectively). There was no significant difference for SO_2_ detection rates across seasons.

### Correlation between outdoor and indoor air pollution

Outdoor PM_2.5_ concentrations were moderately correlated with median indoor PM_2.5_ concentrations that were measured in four to five households in each village (Spearman r_s_ = 0.41, p < 0.0001). Outdoor NO_2_ concentrations were also moderately correlated with median indoor NO_2_ concentrations (r_s_ = 0.43, p < 0.0001). However, a relatively weaker but non-significant correlation was found between outdoor and indoor BaP concentrations (r_s_ = 0.21, p = 0.06).

### Determinants of outdoor pollutants

Of all of the variables considered in the mixed models, coal type from the local coal mine, the percentage of homes in each village with chimney stoves, and season were identified as common factors contributing to outdoor PM_2.5_, BaP, and NAP (Table 4). For NO_2_ only, the average indoor concentration was identified as a contributing factor to its corresponding outdoor concentration (GMR = 1.01, p < 0.05). Coal type and the use of fire pits for cooking were associated with outdoor SO_2_ detection rates.

**Table 4:** Significant determinants of outdoor air concentrations from mixed modeling.

	ln-PM_2.5_			ln-BaP			ln-NAP			SO_2_				ln-NO_2_		
	β	95% CI	GMR^a^	β	95% CI	GMR	β	95% CI	GMR	β	95% CI		OR	β	95% CI	GMR
**Coal type**																
Smokeless coal	Ref		1.00	Ref		1.00	Ref		1.00	Ref		1.00				
Smoky coal	0.31	− 0.31, 0.93	1.36	1.02	0.29,1.76	**2.79**	0.89	0.04,1.75	**2.44**	− 0.85	− 1.58, − 0.12	**0.43**		Not included		
**Proportion of chimney stoves in a village**																
≤ 50%	Ref		1.00	Ref		1.00	Ref		1.00							
> 50%	− 0.02	− 0.39, 0.35	0.98	0.40	0.01, 0.79	**1.49**	0.06	− 0.53, 0.64	1.06		Not included			Not included		
**Season**																
Autumn	Ref			Ref		1.00	Ref		1.00					Ref		1.00
Spring/summer^b^	0.19	− 0.18, 0.56	1.21	0.52	0.21, 0.84	**1.69**	0.29	− 0.14, 0.71	1.33		Not included			− 0.04	− 0.21, 0.14	0.96
Winter	0.62	0.25, 0.99	**1.86**	0.38	0.04, 0.72	**1.46**	1.15	0.65,1.65	**3.17**					0.17	0.00, 0.33	**1.18**
**Population, per 1,000**																
	0.46	− 0.04, 0.95	1.58	Not included			Not included			Not included				Not included		
**Use firepit to cook, 10%**																
Not included				Not included			Not included			0.20	− 0.31, 0.71		1.22	Not included		
**Averaged indoor NO**_**2**_**, µg/m**^**3**^																
Not applicable				Not applicable			Not applicable			Not applicable				0.010	0.006, 0.014	**1.01**
**Coal deposit**																
20,27	Ref			Ref		1.00	Ref		1.00	Not included				Ref		1.00
1, 2, 4, 7, 8	− 0.17	− 0.79, 0.44	0.84	− 0.22	− 0.85, 0.41	0.80	− 0.16	− 0.95, 0.63	0.85					0.18	0.01, 0.35	**1.20**
9	− 0.74	− 2.13, 0.66	0.48	− 0.16	− 0.91, 0.59	0.85	− 0.04	− 1.17,1.08	0.96					0.51	0.21, 0.81	**1.66**
10	− 0.11	− 0.98, 0.77	0.90	0.08	− 0.81, 0.96	1.08	−	−	−					0.31	− 0.01, 0.62	1.36
LS	− 1.40	− 2.88, 0.09	0.25	− 0.81	− 1.61,− 0.01	**0.44**	− 0.38	− 1.53, 0.76	0.68					0.15	− 0.16, 0.46	1.16
12, 13, 14, 38	− 0.18	− 0.84, 0.48	0.83	− 0.30	− 0.94, 0.34	0.74	0.59	− 0.31,1.50	1.81					0.21	− 0.01, 0.44	1.24
16, 17, 19	− 0.32	− 0.95, 0.30	0.72	− 0.48	− 1.12, 0.17	0.62	0.04	− 0.79, 0.86	1.04					0.08	− 0.10, 0.25	1.08
**Intraclass correlation**																
Between villages		1			1			0.86			1				1	
Reference value^c^	3.24 ln-µg/m^3^			0.97 ln-ng/m^3^			5.38 ln-ng/m^3^			–				3.67 ln-µg/m^3^		

*NAP* naphthalene, *BaP* Benzo(a)pyrene, *SO*_*2*_ sulfur dioxide, *NO*_*2*_ nitrogen dioxide.

^*a*^*GMR* = geometric mean ratio = GM (estimate)/GM (reference) = Exp (β), number is bold if β is significantly different from zero (p < 0.05). ^b^Merged due to few outdoor measurements in summer. ^c^Reference value represents log transformed value for the reference model entry.

Burning smoky coal and having > 50% of households with chimneys was associated with higher levels of outdoor BaP (GMR = 2.79, p < 0.05 for smoky coal compared to smokeless coal; GMR = 1.49, p < 0.05 for villages having > 50% of households with chimneys compared to those having ≤ 50% of households with chimneys) when adjusted for the variables shown in Table 4. However, using smoky coal was associated with a lower outdoor SO_2_ detection rate (OR = 0.43, p < 0.05). Outdoor PM_2.5_ (GMR = 1.86, p < 0.05), BaP (GMR = 1.46, p < 0.05), and NAP (GMR = 3.17, p < 0.05) concentrations were significantly higher in winter, and BaP concentrations (GMR = 1.69, p < 0.05) were higher in spring and summer compared to the corresponding pollutant levels in autumn. Outdoor PM_2.5_ and NAP did not vary significantly by coal deposit, but BaP levels in LS site (GMR = 0.44, p < 0.05) were significantly lower than at other sites. For coal deposits located in northeast Xuanwei, NO_2_ levels for the smoky coal deposits (i.e. 1,2,4,7,8 and 9) were significantly higher compared to the smokeless coal deposits (i.e. 20, 27).

In villages with reference entry levels, the log-transformed values for outdoor PM_2.5_, BaP, NAP, and NO_2_ were 3.24 ln-µg/m^3^, 0.97 ln-ng/m^3^, 5.38 ln-ng/m^3^, and 3.67 ln-µg/m^3^, respectively.

## Discussion

The Global Burden of Disease project (GBD) found that household solid fuel use accounted for 12% of ambient PM_2.5_ globally in 2010^20^, with higher contribution in China (19% in 2013) and India (24% in 2015)^21,22^. Exposure assessment for the GBD also showed substantial exposures occurring in rural areas^23^. Potential sources of rural ambient air pollution may be from households using solid fuels for cooking and heating, from nearby urban and rural sources, and from secondary pollutants at intercontinental scales^8^.

Only a handful of studies have reported ambient concentrations in rural areas, as health-damaging air pollution has been considered a largely urban phenomenon^8^. Our study revealed high levels of outdoor air pollution in a rural area of China with a high incidence of lung cancer. The 24-h geometric mean outdoor PM_2.5_ concentrations in villages of the two rural counties, 51.6 µg/m^3^ in Xuanwei and 40.2 µg/m^3^ in Fuyuan, are almost two-fold that of the outdoor PM_2.5_ guideline value set by the World Health Organization (25 µg/m^3^)^3^. Further, measured PM_2.5_ concentrations were similar to the population-weighted average exposure to PM_2.5_ (52 µg/m^3^) in China^24^, higher than those in rural areas in Hong Kong (24.9–30.0 µg/m^3^)^25^, and comparable to levels at suburban sites reported in an earlier study in Guangdong, Wuhan, Lanzhou and Chongqing, China (GM: 39–94 µg/m^3^)^26^.

The geometric means of outdoor BaP were 10.5 and 8.9 ng/m^3^ in Xuanwei and Fuyuan, respectively, which were up to 105 times as high as the background levels in rural areas of the United States reported by the Agency for Toxic Substances and Disease Registry^27^. Both outdoor BaP and NAP in this rural area of China were much higher than the levels found in the rural Cantabria region in Spain (0.15 ng/m^3^ for BaP and < 0.01 ng/m^3^ for NAP)^28^. In Xuanwei and Fuyuan, the combined outdoor BaP and NAP levels were five-fold and two-fold higher than rural air levels in central Taiwan, respectively (1.5 ng/m^3^ for BaP and 223 ng/m^3^ for NAP)^29^. Outdoor NAP levels in our data were even higher than the highest NAP exposure category among occupationally exposed U.S. Air Force personnel^30^. Average BaP levels exceeded the 24-h criterion of the Chinese national standard (2.5 ng/m^3^) by a factor of two^31^.

Varying from 85.1 to 118.5 across all coal deposits, the arithmetic mean outdoor NO_2_ concentrations in our study area were ~ 98 µg/m^3^, two to ten times as high as those found in a rural area in India (4.7–9.6 µg/m^3^)^32^ and an industrial city in Turkey (14.0–57.5 µg/m^3^)^33^.

Outdoor SO_2_ detectable concentrations in our study were negligible compared with the levels measured in a rural area in India (2.5–5.2 µg/m^3^)^32^ and an industrial city in Turkey (5.9–41.2 µg/m^3^)^32,33^. This is consistent with the finding that the rural residents used low sulfur coals in this study area^34^. However, using smoky coal was associated with a significantly lower outdoor SO_2_ detection rate than using smokeless coal (%Detect: 21.6 vs 50%), which is also consistent with both the observations of indoor SO_2_^14^ and in a coal composition analysis^34^.

In South Asia, regional concentrations of ambient PM_2.5_ derived from household cooking with solid fuels in 2010 was 8.6 µg/m^3^, which contributed 12% to ambient PM_2.5_ levels globally^20^. In India, HAP contributes 22–52% to ambient air pollution and 24.2% to ambient PM_2.5_ in rural India^22,35^. Although our study was not designed to measure emissions at the outlet of household chimneys, we calculated the ratio of outdoor levels over the average indoor concentrations measured in each village visited in Xuanwei and Fuyuan. The outdoor/indoor (O/I) ratios were ~ 10 to 30% for PM_2.5_^12^, ~ 20 to   ~ 60% for BaP^13^, and ~ 70 to 80% for NO_2_^14^ depending on the various household solid fuels that were used. It is not surprising that NO_2_ presents a higher O/I ratio due to the higher penetration rates of gaseous pollutants. The higher O/I ratio for BaP compared to PM_2.5_ may imply other outdoor BaP sources, such as from emissions from other households in the village. Taken together, these results suggest that indoor air pollution due to coal combustion in households is a significant source of outdoor air pollution in rural villages.

Ventilated stove and fuel use in rural settings may have different effects on indoor, outdoor air pollution and personal exposures. A previous study of Xuanwei smoky coal use assessed the long-term health benefits of converting from unvented stoves to either stoves with chimneys or portable stoves (which were intended to be lit outdoors before being carried inside for use), and observed reduced indoor air exposures and lung cancer risks^18,19^. This study is the first to evaluate outdoor air pollution in this rural area that has a high incidence of lung cancer. Installing chimneys to simply discharge pollutants from inside homes to the outdoors may increase outdoor concentrations and as a result not sufficiently reduce the overall exposure to carcinogens; therefore, removal of the pollution source by moving populations up the “energy ladder” towards the use of cleaner fuels (e.g. biogas and electricity) should be strived for.

Meteorological factors may affect the spreading of primary pollutants and the formation of secondary pollutants in rural villages^36^. A study conducted in an urban area of Hong Kong found that temperature, humidity, and solar irradiation played a vital role in the variation of the O/I ratio, which increased with upward changes of these weather parameters^37^. However, in the current study, seasonal category was found to more accurately predicate the outdoor exposure than meteorological factors.

Limitations of this study include small sample size and availability of a background monitoring spot in this rural area.

This study showed that outdoor air pollution in a rural region of China with a high incidence of lung cancer was associated with coal type and the proportion of ventilated stoves in a village. These findings suggest that the further reduction of adverse health effects in rural villages from the indoor burning of coal, will most likely require the use of stoves that reduce environmental exhaust, and ultimately the replacement of coal with cleaner fuel types.

## Methods

### Study design and air pollution measurements

The exposure assessment study design and population have been described in detail elsewhere^12^. We reported here the part of outdoor air measurement. Briefly, outdoor air measurements were taken between August 2008 and June 2009 from 29 selected villages in Xuanwei and Fuyuan as part of a large case–control study of lung cancer, and a cross-sectional molecular epidemiological study. Villages were selected to represent all major geological coal deposit areas based on a local geologic analysis of coal-type in Xuanwei and Fuyuan counties. In each selected village, four or five households were selected to conduct personal and indoor exposure measurements, while outdoor air measurements were conducted in a central location within each village in phase I. In phase II, approximately half of the villages (n = 16) and households were visited for a second round of repeated measurements 2–9 months later.

### Village background information

Background demographic information for each village was collected through an in-person interview with either a local doctor or the village head. Global Positioning System (GPS) coordinates were recorded on-site. Survey information included: house types and ventilation, fuels used for cooking and heating, stove types, main coal mines providing coal to the village, village altitude, total population, land area in km^2^, percentage of households having a television set, average household income, and presence of nearby industries. Distribution of some variables were shown in Table 1.

### Outdoor sample collection and analysis

An ambient air monitoring station was installed at a central location within each village away from any direct sources of emissions (e.g. chimneys). The air monitoring methods that were used were similar to those used to measure indoor air pollution and are described in detail^12,13,15^. In brief, samples of fine PM and associated particle phase PAHs were collected on 37 mm Teflon filters using a cyclone with an aerodynamic cut-off of 2.5 µm (model BGI, GK 2.05SH) at a flow rate of 3.5 L/min (± 20%). XAD-2 sorbent tubes were used to measure gas phase PAHs at an air flow rate of 100 mL/min. Particulate mass was measured by pre- and post-weighing of the filters in an environmentally-controlled weighing room using a microbalance at 1 µg accuracy. PAH extraction from the Teflon filters and the XAD-2 sorbent tubes was performed using the US EPA 3540C-1996 (Soxhlet extraction) method to determine concentrations of 16 PAHs by a gas chromatograph connected to a mass spectrometer (Shimadzu QP2010 Plus). Passively diffusing OGAWA badges were deployed to collect measurements of NO_2_ and SO_2_. Flow injection analysis and ion chromatography were used to determine the concentrations of NO_2_ and SO_2_, respectively. In addition, a weather station (WeatherLink Wireless Vantage Pro2) was deployed to record meteorological factors (e.g. temperature, wind speed, humidity, rainfall amount). Two sequential 24-h outdoor air measurements were conducted in each village in parallel with indoor and personal measurements. For quality control purposes, field blank and 13 duplicate filter samples as well as duplicate OGAWA badges (~ 10%) were collected. More than 97% of field blank filters reported non-detect PAHs. The coefficient of variation of the duplicate samples was 13% for PM_2.5_, 25% for BaP, 38% for NAP, and 27% for NO_2_, respectively. The percentage agreement in detect vs non-detect of the duplicate SO_2_ samples was 90%.

### Statistical analyses

Normal probability plots indicated that the measured values could be best described by a log-normal distribution; therefore, outdoor air pollution concentrations were natural log-transformed to approximate normal distributions for the statistical analyses that were conducted. Outdoor measurements were summarized as arithmetic means (AM), geometric means (GM), and geometric standard deviations (GSD) by coal deposit. Meteorological factors are summarized as AM, standard deviations (SD), and medians. Spearman correlations were calculated between concentrations of outdoor and indoor pollutants. Analysis of variance (ANOVA) and Tukey Honestly Significant Difference (HSD) testing was performed on log-transformed values to assess differences between coal deposits, fuel types, stove ventilation, and season. Due to the large proportion of undetectable values of SO_2_ measurements (73.9%), detection rate (%Detect) was calculated for each coal deposit and overall region. Linear mixed effect models were used to identify variables which may be associated with outdoor PM_2.5_, BaP, NAP, and NO_2_, while a mixed effects logistic model was used for SO_2_. Villages were assigned random effects with a variance-components covariance structure. Multiple variables were considered for inclusion as fixed effects including coal deposits, season (winter, spring, summer, autumn), proportion of ventilated stoves (i.e. chimneys) in a village, meteorological factors, proportion of solid fuel types in a village to heat rooms or cook, proportion of stove types used in a village, type of coal mines (i.e. smoky coal or smokeless coal), altitude, village area, population size, nearby industry, average indoor concentration of the pollutant measured in a village, average income and other surrogates of socioeconomic status such as proportion of households having a TV set. Inclusion of variables in the final model was based on the combination of their influence on the Akaike information criterion (AIC) score. The linear mixed effect model can be expressed with the following equation:

$${y}_{ijf}={\mu +\beta }_{1}{x}_{1}+{\beta }_{2}{x}_{2}\dots {\beta }_{n}{x}_{n}+{{b}_{1}I}_{i}+{b}_{2}{J}_{ij}+{\varepsilon }_{ijf}$$

where *y*_*ijf*_ represents the natural log-transformed value of outdoor pollutant concentration being modeled for coal deposit *i* in village *j* on day *f*; *μ* represents the intercept value (i.e., the log-transformed value for the reference group); *β*_1_ through *β*_n_ represent the fixed effect variable coefficients for variables *x*_1_ through *x*_n_ ; b_1_*I*_*i*_ represents the coefficient for coal deposit *i*; b_2_*J*_*ij*_ represents the random effect coefficient for village *j* from coal deposit *i*; and *ε*_*ijf*_ represents the error for village *j* in coal deposit *i* on day *f*.

All analyses were carried out using SAS/STAT software, version 9.4 of the SAS System for Windows. Copyright © 2016 SAS Institute Inc. SAS and all other SAS Institute Inc. product or service names are registered trademarks or trademarks of SAS Institute Inc., Cary, NC, USA.

The study protocol was approved by the institutional review boards of the National Cancer Institute and China National Environmental Monitoring Center. All participants provided written informed consent prior to participating in the study. This study was conducted in accordance to the World Medical Association Declaration of Helsinki’s recommendations for human subject protection.

### Consent for publication

The content of this publication does not necessarily reflect the views or policies of the Department of Health and Human Services, nor does the mentioning of trade names, commercial products, or organizations imply endorsement by the U.S. Government.

## Data Availability

All data generated or analyzed during this study are included in this published article.
